# Performance of EuroSCORE II in octogenarians with comorbidities undergoing aortic valve replacement

**DOI:** 10.1186/1749-8090-10-S1-A57

**Published:** 2015-12-16

**Authors:** Hernandez-Vaquero Daniel, Rocio Diaz, Ruben Alvarez-Cabo, Carlos Morales, Jacobo Silva

**Affiliations:** 1 Cardiac Surgery Department, Central University Hospital of Asturias, Oviedo, Spain

## Background/Introduction

EuroSCORE II is being used to select high-risk patients who are candidates for transcatheter procedures. The majority of these patients are octogenarians with comorbidities. However, to date, no studies have evaluated the performance of EuroSCORE II in octogenarians with renal insufficiency or left ventricular dysfunction.

## Aims/Objectives

To know the performance of EuroSCORE II in octogenarian patients with severe renal insufficiency and ventricular dysfunction who undergo traditional aortic valve replacement.

## Method

All octogenarians who underwent aortic valve replacement between 2009 and 2015 in our center were analyzed. Creatinine clearance was assessed using Cockroft- Gault formula as recommended for authors of EuroSCORE II. Severe renal insufficiency was considered when the creatinine clearance was <50 ml/min as indicated in EuroSCORE II. Ventricular dysfunction was considered when the left ventricular ejection fraction was <50%. Discrimination was evaluated by the area under the receiver operating curve (AROC) and for calibration value of p for Hosmer-Lemeshow test and risk adjusted mortality ratio (RAMR) were calculated.

## Results

482 octogenarian patients underwent traditional aortic valve replacement during the study period. Between these patients, 120 (24,9%) had severe renal insufficiency and 78 (16,2%) had left ventricular dysfunction. 18,3% of patients with severe renal insufficiency and 17,9% of patients with ventricular dysfunction died. Discrimination was very good in both subgroups with an AROC of 0,88 and 0,85 for patients with renal insufficiency and left ventricular dysfunction respectively. However, calibration was poor. Hosmer-Lemeshow test showed a value of p = 0,02 and 0,075 for patients with renal insufficiency and ventricular dysfunction and RAMR showed much more observed mortality than expected (RAMR = 18,3/8,9 = 2,05 and 17,9/11,4 = 1,6) Logistic EuroSCORE showed the best calibration accuracy.

## Discussion/Conclusion

Using the formula recommended for EuroSCORE II, 25% of the octogenarians undergoing aortic valve replacement have severe renal insufficiency. In these patients and in those with left ventricular dysfunction EuroSCORE II is able to accurately distinguish patients at high surgical risk. However, EuroSCORE II underestimates the real risk of octogenarians with these risk factors and the logistic EuroSCORE has the best calibration accuracy.

